# *In vitro* assessment of Neuronal PAS domain 2 mitigating compounds for scarless wound healing

**DOI:** 10.3389/fmed.2022.1014763

**Published:** 2023-02-01

**Authors:** Adam Clements, Yoichiro Shibuya, Akishige Hokugo, Zachary Brooks, Yvonne Roca, Takeru Kondo, Ichiro Nishimura, Reza Jarrahy

**Affiliations:** ^1^Regenerative Bioengineering and Repair Laboratory, Division of Plastic and Reconstructive Surgery, Department of Surgery, David Geffen School of Medicine, University of California, Los Angeles, Los Angeles, CA, United States; ^2^Weintraub Center for Reconstructive Biotechnology, UCLA School of Dentistry, University of California, Los Angeles, Los Angeles, CA, United States

**Keywords:** wound healing, skin, scarring, circadian clock, fibroblast

## Abstract

**Background:**

The core circadian gene Neuronal PAS domain 2 (*NPAS2*) is expressed in dermal fibroblasts and has been shown to play a critical role in regulating collagen synthesis during wound healing. We have performed high throughput drug screening to identify genes responsible for downregulation of *Npas2* while maintaining cell viability. From this, five FDA-approved hit compounds were shown to suppress *Npas2* expression in fibroblasts. In this study, we hypothesize that the therapeutic suppression of *Npas2* by hit compounds will have two effects: (1) attenuated excessive collagen deposition and (2) accelerated dermal wound healing without hypertrophic scarring.

**Materials and methods:**

To test the effects of each hit compound (named Dwn1, 2, 3, 4, and 5), primary adult human dermal fibroblasts (HDFa) were treated with either 0, 0.1, 1, or 10 μM of a single hit compound. HDFa behaviors were assessed by picrosirius red staining and quantitative RT-PCR for *in vitro* collagen synthesis, cell viability assay, *in vitro* fibroblast-to-myofibroblast differentiation test, and cell migration assays.

**Results:**

Dwn1 and Dwn2 were found to significantly affect collagen synthesis and cell migration without any cytotoxicity. Dwn3, Dwn4, and Dwn5 did not affect collagen synthesis and were thereby eliminated from further consideration for their role in mitigation of gene expression or myofibroblast differentiation. Dwn1 also attenuated myofibroblast differentiation on HDFa.

**Conclusion:**

Dwn1 and Dwn2 may serve as possible therapeutic agents for future studies related to skin wound healing.

## Introduction

Skin wound healing following injury, surgical procedure, burn, or from systemic disease remains a common clinical problem that requires reliable wound management. The goal of wound management is to fully restore the protective function of the skin as quickly as possible and to optimize appearance (1). Hypertrophic scars (HTS) result from an abnormal fibrous wound healing process in which tissue repair and regeneration-regulating mechanism control is lost (2). The formed scar is often raised and discolored in comparison to surrounding structures, exhibiting abnormally high levels of collagen in a different ratio of collagen types to normal skin (3). Regulation of these collagen types offers a possibility to reduce the prevalence of HTS following surgical operations. As such, the ability to impact HTS formation remains a promising avenue for improving clinical treatment of surgical wounds. These wounds, in addition to being aesthetically undesirable, also result in a decrease in functionality of the skin. With the overexpression of collagen, scarred skin exhibits lower elasticity and strength when compared to healthy counterparts (2). Furthermore, the presence of HTS on facial tissues can result in psychological damage to one’s self-image and dissatisfaction with life. This often results in additional repair procedures aimed at reducing the prevalence of the scar tissue, costing the patient thousands of dollars out-of-pocket. Therein, the current study aims to mitigate the formation of these scars while simultaneously accelerating the overall wound healing process.

In the normal wound healing process, skin recovers through a series of three distinct phases, each comprising their own timeline (4). The inflammatory phase lasts first and continues for 48–72 h. During this time, specific cell types, namely, neutrophils, leukocytes, and macrophages, migrate toward the wound site in response to cytokine gradients generated from the activation of the coagulation cascade. These migratory cells induce the expression of pro-inflammatory cytokines to augment the inflammatory response and recruit non-specific immune cells to provide early detection of potential pathogens within the area. A key transitional marker between the inflammatory phase and the proliferative phase is the change in macrophage activity. These macrophages are formed *via* differentiation of local monocytes and start as M1 macrophages, responsible for production of pro-inflammatory cytokines, mediate resistance to pathogens, and exhibit antimicrobial properties. However, at the initiation of the proliferative phase of wound healing, these M1 macrophages change to M2 macrophages, responsible for anti-inflammatory mediators and aid in the regeneration of the extracellular matrix (ECM). This phase is also characterized by the formation of structural elements of the ECM designed to support vascular growth *via* expression of procollagen, elastin, and hyaluronic acid. Not only that, but granulation tissue and epithelial cells begin to fill the wound *via* cellular migration and mature collagen fibers are generated to provide structural support. This phase can last several weeks before transitioning to the final maturation phase of wound healing, which includes tissue strengthening and further progression of the scarring process (4).

Circadian clock genes are responsible for the maintenance of cellular metabolism within the body’s tissues, determining the way in which cells respond to changing environmental stimuli (5). In general, circadian rhythms dictate not only sleep, hunger, and energy levels throughout the day, but also play a role in the ability for individual cells or tissues to maintain their metabolic cycles. Specifically, the core circadian clock is controlled by the suprachiasmatic nuclei (SCN), located within the hypothalamus (6). The circadian locomotor output cycles kaput (*Clock*), Neuronal Pas Domain 2 (*Npas2*), and aryl hydrocarbon receptor nuclear translocator-like protein 1 (*Arntl, Bmal1*) genes are responsible for producing the transcription factors used to maintain the core circadian clock by working together to induce expression in the period (*Per2*) and cryptochrome (*Cry1* and *Cry2*) genes. This is done through the formation of heterodimers between the protein products of these genes (Clock-Bmal1 and Npas2-Bmal1, respectively), which then regulate *Per2* and *Cry1* at both the organismal and cellular level to control the 24-h sleep/wake cycle (7). Previous studies have shown that skin wounds in mice wounded during the circadian rest period healed less quickly than those wounded during the active period, and human burn injuries incurred during the night healed more slowly than wounds acquired during the day (8). This phenomenon is believed to be due to the strong correlation between circadian cycles and activity of different immunological cell types (9). Leukocytes, as mentioned previously, are important for preliminary wound healing mechanisms and have been shown to fluctuate in response to circadian activity (10). The level of recruited leukocytes to specific tissues has been shown to increase during periods of active metabolic process as mediated by the expression of chemokines by endothelial cells (10). As such, these circadian-mediated levels of circulating cells are thought to impact the mechanisms by which wounds heal, as the lack of adequate cell types in a wound site produces an imbalance of pro-inflammatory, anti-inflammatory, ECM generating, and ECM degrading signals that are essential for proper wound healing to occur. As a result, these fluctuations in circadian activity influence the manner in which wounds heal through indirect manipulation of contributory cells and serve as a promising avenue for further research.

In our previous study, we reported that mice lacking the *Npas2* gene (*Npas2-/-*) exhibit accelerated dermal wound healing mechanisms in comparison to those with fully functional circadian rhythm genes and showed increased cellular migration and contraction *in vitro* (11). In addition, we demonstrated that *Npas2*-/- mice showed a lower level of ECM deposition on the surface of the biomaterials compared with wild-type mice when the biomaterials were exposed to the bone tissue. The collagen structure was less visible in tissue remnants on the biomaterials recovered from *Npas2-/-* mice (12). Taken together, we seek to balance the beneficial effects of accelerated wound closure with the detrimental effects of excessive collagen deposition caused by lacking circadian.

Our hypothesis is that therapeutic suppression of *Npas2* by hit compounds will lead to two outcomes: (1) attenuated excessive collagen deposition and (2) acceleration of wound healing. The objective of this *in vitro* study is to determine the extent to which hit compounds that mitigate *Npas2* gene expression identified by a high throughput drug screening can regulate collagen synthesis and cellular bioactivity on fibroblast.

## Materials and methods

### Statement of animal use

All protocols for animal experiments were approved by the University of California Los Angeles Animal Research Committee (ARC# 2003–009) and followed the Public Health Service Policy for the Humane Care and Use of Laboratory Animals and the UCLA Animal Care and Use guidelines. The animals were fed a regular rodent diet and provided water *ad libitum*. They were maintained in regular housing conditions with 12-h light/dark cycles in the Division of Laboratory Animal Medicine at UCLA.

### High throughput drug screening

At the Molecular Screening Shared Resource at UCLA, a drug library of 1,120 FDA-approved compounds were screened with two different assays to identify hit compounds with wound healing properties. Hit compounds involved in the modulation of murine dermal fibroblast *Npas2* expression were identified using high-throughput screening of gene activity levels. Dermal fibroblasts were isolated from mice engineered to carry the *LacZ* reporter gene in the *Npas2* allele. *LacZ* reporter gene activity has previously been shown to accurately correlate with endogenous *Npas2* expression (11). The cells were cultured in growth medium containing Dulbecco’s Modified Eagle’s medium (DMEM, Life Technologies Corp, Carlsbad, CA, USA) with 10% fetal bovine serum (FBS, Life Technologies Corp, Carlsbad, CA, USA) and 1% penicillin/streptomycin (PS, Life Technologies Corp, Carlsbad, CA, USA). Using 384-well plates (Greiner Bio-One, Monroe, NC, USA) and a pin tool (Beckman Coulter, Brea, CA, USA), each well was filled with 25 μl non-phenol red DMEM (Life Technologies Corp, Carlsbad, CA, USA), which contained 10% FBS and 1% PS, and 50 nL of 1,120 high-purity chemical compounds from an FDA-approved drug library available at Molecular Screening Shared Resource at UCLA, giving a final concentration of 1 μM per individual compound being tested. Since each well contained a single compound, this experiment gave evidence of how each molecule was able to influence the *Npas2* gene expression. Cells were added to each well (1,500 cells per 25 μl) and incubated at room temperature for 1 h, followed by a 48 h incubation at 37°C and 5% CO_2_. To measure *Npas2-LacZ* expression, β-galactosidase activity was measured using a Beta-Glo Assay System (Promega, USA). The *Npas2-LacZ* expression data were uploaded to an online data analysis tool (CDD Vault, Collaborative Drug Discovery Inc., Burlingame, CA, USA), on which data were normalized and the *Z*-factor was calculated.

The possibility exists that some compounds that suppress *Npas2* might be false positives due to cytotoxicity that leads to cell death or suppresses growth in cells that have not been suppressed *Npas2*. In order to prevent this problem, separate high-throughput screening for cell viability was conducted using the same FDA-approved drug library. A commercially available human dermal fibroblast cell line (CCD-1122Sk, ATCC; 3,000 cells per 25 μL) was applied on OrisTM Pro Cell Migration Assay 384-well plate (Platypus Technologies, Fitchburg, WI, USA), which has a water-soluble biocompatible gel that creates a center cell-free detection zone for cell migration in each well. After plating the cells, the plates were centrifuged at 200 rotations per minute (RPM) for 5 min. After a 1-h incubation at room temperature for cell attachment, the compounds were added to low-FBS medium using a 250 nL pin tool and incubated at 37°C in the CO_2_ incubator. After 48 h of incubation, 25 μL of staining solution (Calcein-AM and Hoechst, Life Technologies Corp, Carlsbad, CA, USA) was added to each well. After another period of centrifugation at 200 x *g* for 5 min, the plates were incubated for 20 min at room temperature, and each well was then imaged by the Micro Confocal High-Content Imaging System (ImageXpress, Molecular Devices, USA). The cells that migrated into the detection zone were counted using a customized computer program (CDD Vaultâ, Collaborative Drug Discovery, USA) and the *Z*-factor was calculated.

### Dermal fibroblast cell culture

In the high throughput drug screening, the use of murine dermal fibroblasts was important for determining the ability of each hit compound to modulate the *Npas2* using *LacZ* gene expression reporter system. However, the use of human dermal fibroblasts (HDFa) was then used to determine how the selected compounds would be able to maintain a high cell viability and biological activities as it relates to clinical implementation. Primary human dermal fibroblasts, adult (HDFa) were obtained from ATCC, USA. Cells were cultured in growth medium including modified DMEM with 10% FBS and 100 units of penicillin per 0.1 mg/ml streptomycin at 37°C and 5% CO_2_ in a humidified incubator. In addition, growth media was supplemented with L-ascorbic acid (AA) (1 mM; Sigma-Aldrich Corp., Saint Louis, MO, USA) as AA has been shown to be essential for the development of a normal, strong, and mature collagen network as well as maintaining its optimal collagenic density (13). Medium changes were conducted every 3 days.

### Cell viability assay

WST-1 test was conducted as an indicator of fibroblast viability after hit compound treatment. HDFa were seeded at a concentration of 2 × 10^3^ cells/well in a 96 well plate. The culture medium was added with various amount of each hit five compounds (0.1, 1, 10 μM) to the well (*N* = 5). These cells were then incubated at 37°C and 5% CO_2_ in a humidified incubator. After 1–3 days incubation, cell proliferation and viability reagent (WST-1, Sigma-Aldrich Corp., Saint Louis, MO, USA) was added and cells were allowed to incubate for 1 h at 37°C. These cells were then shaken for 1 min and the absorbance of these cells was determined at 450 nm using a plate reader (SYNERGY H1, BioTek, Calabasas, CA, USA). Statistical analysis was performed using GraphPad Prism by two-way ANOVA and Dunnett’s tests, using the Vehicle group as the point of comparison. This experiment was conducted *via* three repetitions.

### Collagen synthesis by picrosirius red staining

Human dermal fibroblasts were seeded in a 24-well plate at 20,000 cells/cm^2^. After cells became confluent, cells were treated with the growth medium with AA and various concentrations (0, 0.1, 1, and 10 μM) of the hit compounds and the negative control was placed in identical conditions without AA supplementation (*N* = 4). On day 14, the cells were fixed with 10% neutral buffered formalin and stained with picrosirius red (PolyScience, USA) for gross collagen visualization. For a quantitative analysis, the stained dye was then eluted in 0.1 N sodium hydroxide from each well, and a plate reader (SYHNERGY H1 plate reader, USA) was used to determine the absorbance at a wavelength of 550 nm. This experiment was conducted *via* three repetitions, with positive controls representing no hit compound treatment with AA supplementation and negative controls representing no hit compound treatment without AA supplementation. Statistical analysis was performed by one-way ANOVA and Dunnett’s tests, using the 0 μM + AA group as the point of comparison.

For the purposes of this experiment, media changes with AA supplementation were conducted every 3 days over a course of 14 days, with each media change coinciding with a simultaneous hit supplementation by a single hit compound at the specified dosage for that treatment group. This method was used to study the effect of each hit compound at the specified concentration throughout the course of normal wound healing. Typically, this experiment would be conducted over 48 h, yet the extension to 14 days allowed for investigation of how these cells would respond in a simulation of multiple phases of wound healing. Specifically, this allowed for investigation of how the development of collagen would progress into the proliferative phase of wound healing, thereby giving a more accurate representation of the level of collagen formation at the time of hypertrophic scar development in healing cutaneous wounds.

### Collagen-related gene expression

One of the purposes of this study is to determine the level of gene activity during the height of collagen formation to serve as an indicator of how healing cutaneous wounds would induce collagen formation during the window in which HTS commonly form. As such, this experiment sought to replicate the time frame during the proliferative phase of wound healing to give a more accurate depiction of how hit compound supplementation would mitigate the excessive collagen expression characteristic of HTS.

Human dermal fibroblasts were seeded in a six-well plate at 20,000 cells/cm^2^. After cells became confluent, cells were treated with the growth medium with AA and various concentrations (0, 0.1, 1, and 10 μM) of the Dwn1 and Dwn2 for 14 days (*N* = 3). Total RNA was extracted from cultured HDFa on experimental day 14 using the RNeasy kit (Qiagen, USA), and were then analyzed for quality and concentration using NanoDrop (Thermo Fisher Scientific, Waltham, MA, USA). cDNA synthesis was carried out using the High-Capacity RNA-to-cDNA™ kit (Thermo Fisher Scientific, Waltham, MA, USA) following the manufacturer’s protocol. Quantitative real-time PCR (qPCR) was carried out using TaqMan primer/probe sets (Thermo Fisher Scientific, Waltham, MA, USA), as per manufacturer instructions, for genes collagen type I (*COL1A1*: Mm0080166_g1, *COL1A2*: Mm00483888_m1) and collagen type III (*COL3A1*: Mm00802300_m1). This experiment was conducted *via* three repetitions, with positive controls representing no hit compound treatment with AA supplementation and negative controls representing no hit compound treatment without AA supplementation. Statistical analysis was performed by one-way ANOVA and Dunnett’s tests, using the 0 μM + AA group as the point of comparison.

### *In vitro* fibroblast-to-myofibroblast differentiation test

To observe the myofibroblast differentiation on HDFa, HDFa were cultured in a six-well plate in the growth medium containing 10 ng/ml recombinant human Transforming Growth Factor Beta 1 (TGF-b1) (R&D Systems, USA) and AA with various concentrations (0, 0.1, 1, and 10 μM) of the Dwn1 and Dwn2 for 3 days (*N* = 3). Total RNA was extracted and cDNA synthesis were carried as described above. The qPCR was carried out using TaqMan primer/probe for a myofibroblast marker, alpha smooth muscle actin (a-SMA) (14) (*ACTA2*: Mm01546133_m1). This experiment was conducted *via* three repetitions, with positive controls representing no hit compound treatment with AA supplementation and negative controls representing no hit compound treatment without AA supplementation. Statistical analysis was performed by one-way ANOVA and Dunnett’s tests, using the 0 μM + AA group as the point of comparison.

### Cell migration assay

Cells were evaluated for their ability to infiltrate a wound site through a scratch test. HDFa were able to proliferate until confluence, then make a scratch line with a micropipette tip. The cells were then able to migrate into the scratch area throughout 48 h. The number of cells that infiltrated the wound site were imaged in 16-h increments to analyze the number of migratory cells (*N* = 6). Once all photographs had been captured, the number of migratory cells was determined using ImageJ software. Graphs were generated for Dwn1 and Dwn2 treatment with each respective therapeutic concentration for the total 48-h treatment. This experiment was conducted *via* three repetitions. Statistical analysis was performed using GraphPad Prism by two-way ANOVA and Dunnett’s tests, using the 0 (vehicle) group as the point of comparison.

## Results

### Identification of hit compounds

Following the drug screening of the different compounds, each of the compound’s *Npas2* downregulation *Z*-score was correlated to their respective cell migration *Z*-score. High magnitude, negative *Z*-scores for *Npas2* were used as indicators of significant gene downregulation, while high cell viability *Z*-scores were used to indicate that fibroblasts being treated remained viable through the course of the experiment, eliminating false positive. To select compounds, those with a *Npas2* gene activity *Z*-score greater than −2 and those with a cell viability Z-score less than 1 was eliminated from consideration. The −2 *Z*-score value indicates an *Npas2* downregulation within the 98th percentile of the all compounds tested and the Z-score of 1 indicates a cell viability in the 84th percentile. Together, these selection criteria were able to weed out all but the 5 selected hit compounds. The remaining five compounds (named Dwn1–5) were then chosen to be used as possible therapeutic agents and were assigned identifying names in order of decreasing cell viability index *Z*-score values. The mechanisms of action for these compounds were then identified and recorded in Table 1. The specific cellular signaling pathways employed by each compound differ, thereby causing different levels of effect to the *Npas2* and thus different levels of collagen deposition in the wound site. The information provided (Table 1) represents the current information available in published literature and may serve as a point of comparison between these hit compounds and other drugs with similar properties.

**TABLE 1:** Identified hit compounds and their mechanism of actions.

Hit compound	Class of molecule	Mechanism of action	Common use
Dwn1	Antagonistic	Inhibitor of the monoamine vesicular monoamine uptake transporters	Used to treat hypertension and psychiatric disorders
Dwn2	Antibiotic	Binds to the Qi site of Cytochrome C Reductase to inhibit the oxidation of ubiquinol to ubiquinone	Anti proliferation of cancer cells
Dwn3	Analgesic/Anti-inflammatory	Inhibitor of phospholipase A1 and cyclooxygenase-2; Calcium activated chlorine channel blocker	Treatment of inflammation, pain, and edema
Dwn4	Conventional Antipsychotic	Occupies dopamine D2 receptor sites in reticular limbic systems of brain to decrease dopamine activity	Antipsychotic used to treat schizophrenia
Dwn5	Psychostimulant	Stimulation of Central Nervous System	Not clinically used

### Cell viability on HDFa treated with hit compounds

**FIGURE 1:**
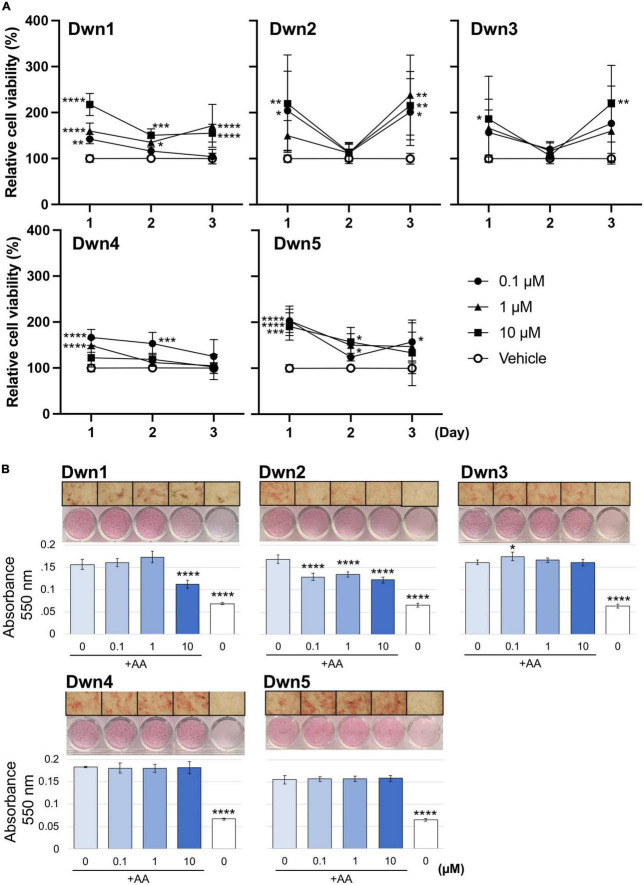
Cell viability and collagen synthesis after hit compound treatment. **(A)** Cellular viability was analyzed using a WST-1 assay, showing the percentage of the relative cell viability under each hit compound compared with vehicle-treated cells (*N* = 5). Analysis consisted of Dunnet’s Test and two-way ANOVA (*P < 0.05, **P < 0.01, ***P < 0.001, ****P < 0.0001; significant difference determined in comparison to vehicle control; bars representative of standard deviation). **(B)** Absorbance values were calculated from spectrophotometer data after picrosirius red staining of human dermal fibroblasts (HDFa). Higher levels of total collagen expression elicited higher absorbance values due to the red staining. Images were taken on D14 and can be seen above each respective graph. The positive control was the 0 μM group cultured with AA supplementation in the growth medium. The negative control was the 0 μM group treated cultured without AA supplementation (*N* = 4). Analysis consisted of one-way ANOVA and Dunnet’s Test (****P < 0.0001; significant difference determined in comparison to 0 μM with AA group; bars representative of standard deviation). The x-axis indicates hit compound concentrations while the black underscore indicates which groups contained AA supplementation.

To determine if the hit compounds were able to attenuate the collagen production *via* actual downregulation of related genes or simply eradication of fibroblasts, a cell viability assay was conducted over 3 days for each hit compound concentration, as shown in Figure 1A. Absorbance values were normalized with respect to the vehicle to show how different hit compounds would be able to maintain a high cell viability compared to the untreated control. This method of characterization allowed for interpretation of how each drug concentration promoted cell survival as a function of time and as a function of concentration. As a result, this assay allowed for initial evaluation of optimal drug concentrations and the point at which compound supplementation became detrimental to cell viability. Compared to the vehicle, each hit compound showed an increase in cell viability for some portion of treatment. Dwn1 showed significant increases for some concentration of hit compound on each day, with 1 and 10 μM treatment groups causing significant cell viability. Dwn2 also showed significant cell viability for all concentrations of hit compounds at day 1 and 3 (0.1 *p* < 0.01, 1 *p* < 0.0001, 10 *p* < 0.001). Dwn3 showed a significant increase with only 10 μM treatment on day 1 and 3. Dwn4 showed an increase in cell viability on days 1 and 2 by the 0.1 μM treatment groups. Dwn5 showed significant increases in cell viability each day for the 0.1 groups. Taken together, these results indicate the ability for each hit compound to induce higher cell viability than the vehicle. Statistical analysis consisted of Dunnet’s Test and two-way ANOVA.

### Overall collagen biosynthesis on HDFa treated with hit compounds

Determination of overall collagen biosynthesis was used as an indication of each compound’s ability to serve as a treatment for the prevention of HTS formation. The significant difference noted between the positive and negative control groups can be attributed to the lack of AA supplementation in the negative control media. The AA has been shown to be significant for the development of strong collagen fibers, with collagen that was formed in medium lacking AA being more prone to instability. For this reason, the overall collagen biosynthesis for the negative control is significantly lower for all groups (Figure 1B). Dwn1 (10 μM) and Dwn2 (0.1, 1, and 10 μM) both displayed a significant collagen deposition in comparison to the AA positive control group. Dwn3 (0.1 μM) showed an increase in overall collagen deposition compared to AA positive control, despite higher drug concentrations of therapeutic agents (1 and 10 μM) resulting in no statistical difference. All concentrations of Dwn4 and Dwn5 showed no significant change in collagen deposition throughout the duration of the experiment.

Since there was only found to be significant collagen mitigation by Dwn1 and Dwn2 following Picrosirius Red staining, these two compounds were considered for gene expression experimentation. The other compounds (Dwn3–5) were eliminated due to the inability to induce significant collagen mitigation following treatment.

### Collagen-related gene expressions on HDFa treated with Dwn1 and Dwn2

**FIGURE 2:**
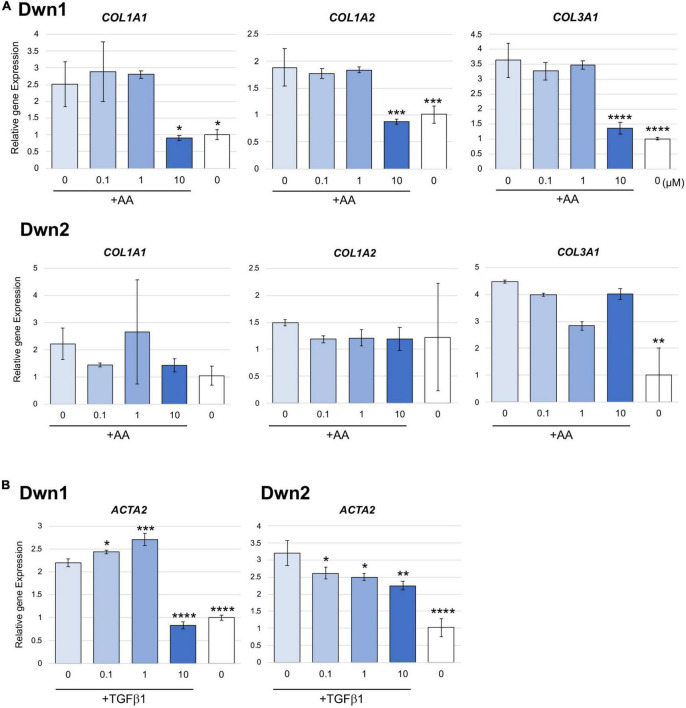
Gene expression for Dwn1 and Dwn2 at various concentrations. **(A)** Figure represents the gene expression of collagen type I (*COL1A1, COL1A2*) and collagen type III (*COL3A1*) on HDFa treated with various dose of Dwn1 or Dwn2 supplemented with AA (*N* = 3). **(B)** Figure represents a-SMA (*ACTA2*) gene expression on human dermal fibroblasts (HDFa) with various dose of Dwn1 or Dwn2 under TGFb1 and AA stimulation (*N* = 3). The graphs represent the average ± STD. The gene expression values analysis consisted of one-way ANOVA and Dunnet’s Test (*P < 0.05, **P < 0.01, ***P < 0.001, ****P < 0.0001).

Relative gene expression of collagen type I (*COL1A1, COL1A2*) and collagen type III (*COL3A1*) were measured on day 14 for Dwn1 and Dwn2 to determine the effect of each hit compound on specific genes. Dwn1 (10 μM) showed a decrease in all gene expression levels (*p* < 0.05, *P* < 0.0001). On the other hand, Dwn2 was not able to significantly downregulate any genes for all treatment concentrations (Figure 2A).

### Myofibroblast differentiation on HDFa treated with by Dwn1 and Dwn2

Relative gene expression of myofibroblast marker gene, *ACTA2* were measured on Day 3. Dwn1 treatment significantly decreased the expression of *ACTA2* on TGFb1-treated HDFa at the dose of 10 μM. All doses of Dwn2 treatment significantly decreased the expression of *ACTA2* on TGFb1-treated HDFa (Figure 2B). These results indicate that Dwn1 and Dwn2 inhibit differentiation of myofibroblasts.

### Cell migration on HDFa treated with Dwn1 and Dwn2

Dwn1 and Dwn2 exhibit a significantly higher level of migratory cells throughout the 48-h treatment when compared to the control group. Dwn1 exhibited significantly higher cell migration for the 0.1 and 1 μM treatment groups. Dwn2 also exhibited higher cell migration for the 0.1 and 10 μM treatment groups.

## Discussion

The field of wound healing has been progressing toward the use of upstream regulators due to their ability to impact multiple levels of the wound healing process with the administration of a single agent. In doing so, these compounds can better modulate cellular behavior in response to injury. This work has direct implications in the healing of surgical wounds, specifically following surgery, as the ability to prevent the formation of HTS will enhance both clinical success and patient experience (15). Patients with HTS following craniofacial surgery not only exhibit poorer clinical success following the procedure, but often undergo subsequent procedures to remove or hide the scar itself (16). In doing so, they risk alterations to the original work, worsening of scar presentation, and damage to surrounding healthy tissue (17). These kinds of scars can cause significant damage to a patient’s psychological wellbeing and can lead to decreased satisfaction with life, an altered perception of body image, and higher rates of post-traumatic stress disorder, alcoholism, imprisonment, unemployment, or marital discord (15). This study has implications across multiple fields of study, primarily focusing on circadian biology and wound healing. By influencing the way in which surgical wounds heal, accelerated wound healing can provide clinical benefits while also mitigating cost. Ranging from physical benefits, including a reduced chance of infection and a greater degree of biofunction following the wound healing process, to the psycho-social benefits of preventing the formation of unwanted scarring, improvements to this field could vastly improve the individual patient experience. By implementing these hit compounds into clinical practice, surgical wounds may be able to heal faster and produce substantial benefits to the patient during the recovery process following surgical procedures.

Recent studies have also established the relationship between circadian rhythm changes and the rate at which dermal wounds heal. The role of specific cell types, including dermal fibroblasts, macrophages, keratinocytes, and subcutaneous adipocytes, all exhibit varying amounts in response to circadian gene activity levels (18). Furthermore, these cell types have been shown to respond not only to the core circadian genes, but also the heterodimer *Bmal1*. Recent studies have linked the depletion of *Bmal1* with the loss of subcutaneous adipose tissue (19) and the accumulation of reactive oxygen species due to the lack of proper circadian control (20). Taken together, these results show how the normal physiology of dermal wounds is impacted by the manipulation of circadian genes, leading to impaired wound healing processes. In addition to *Bmal1*, this same phenomenon is seen in mice with altered multifunctional nuclear protein NONO or Per genes (21). In addition to direct manipulation of the genes responsible for maintaining circadian control, the disruption of normal 24-h circadian cycles due to exposure of test subjects to light during normal times of darkness has also been shown to delay wound healing (22). These disruptions to normal physiological processes work by altering the way in which specific cell types respond to the changing stimuli of a healing wound by changing the time in which normal skin processes occur. Activities that normally occur in the day, such as sebum production and collagen fibril generation, begin to interfere with activities that occur mostly at night, such as DNA repair, cell proliferation, and skin barrier recovery (23). Additionally, the manipulation of circadian activity has been shown to alter keratinocyte physiology (24), causing a delay in normal cellular activity in a wound site. Further studies have identified circadian control in larger areas of dermal physiology, such as the adequate hydration of the epidermis and stratum corneum (25), rather than the specific cell types present. In summary, although there is a few evidence in the current literature of *Npas2-*specific control of dermal wound healing (11, 26), there is an extensive publication about the way elements of the circadian system interact with skin physiology to impact wound healing. As such, this study puts forth a novel mechanism by which the circadian system is able to impact cutaneous wounds in order to accelerate dermal wound healing while focusing on the mitigation of excess collagen deposition.

Due to *Npas2* being deleted from subjects with functioning *Clock* genes, wounds were able to be evaluated in peripheral tissues with absent circadian control at a cellular level yet in-tact circadian control of the sleep/wake cycle, thereby avoiding the complication of delayed wound healing due to entirely absent circadian control. These results coincide with prior findings, which have shown that injuries occurring in the active phase of cellular metabolism have a significantly faster recovery rate than injuries sustained during the dormant phase, likely due to heightened immunological activity during active phases of cellular metabolism (27). For example, hamsters trained to operate on a regimented 24-h circadian rhythm exhibited an increase in wound healing time depending on when the injury applied. Those whose injuries were inflicted during daylight hours healed significantly faster when juxtaposed with those who were injured during night hours (28). Since hamsters are nocturnal and typically have higher levels of cellular activity at night, the ability to induce faster wound healing during the day demonstrates how the absence or lowering of peripheral circadian control may accelerate the wound healing process. With this, the ability to cause *Npas2* suppression allows for a 24-h active cellular metabolism mechanism in peripheral tissue, thereby accelerating wound healing in mouse dermal tissues.

While this is not the only circadian gene that contributes to the circadian cycle at a cellular level, *Npas2* is unique in that it is a paralog of *Clock* in that both are basic-helix-loop-helix (b-HLH) transcription factors able to dimerize with *Bmal1* yet is the only one of the two genes *(Npas2* and *Clock)* expressed in murine dermal fibroblasts (11). Thus, there is not a Clock-Bmal1 heterodimer present to interact with *Per* and *Cry*, thereby allowing *Npas2* to regulate cellular metabolism in isolation. In addition, the level of *Npas2* activity has been shown to be higher in peripheral tissues, rather than in the SCN directly, supporting the theory that *Npas2* may play a larger role in the regulation of peripheral tissue circadian cycles (29). These characteristics allow for *Npas2* to be studied directly rather than in conjunction with *Clock*, thereby offering insights into the mechanism of action *Npas2* exhibits in a wound healing model and how this gene is capable of regulating collagen deposition.

Through the high-throughput screening of 1,120 FDA-approved compounds, the *Npas2* gene has been shown to be downregulated when treated with any one of five different hit compounds (Table 1). We have put forward a novel approach to downregulation of the core circadian clock gene, *Npas2*, to accelerate fibroblast bioactivities and reduce the amount of collagen expressed during dermal wound healing. We hypothesized that downregulation of collagen synthesis genes by different hit compounds will accelerate dermal wound healing with attenuating the formation of HTS. Through the *in vitro* experiments with all five hit compounds identified, Dwn1 and Dwn2 have each been shown attenuated collagen synthesis (Figure 1A). Moreover, the selected hit compounds maintained high cell viability and did not have any cytotoxicity (Figure 1B), yet all have different mechanisms of action. The mechanisms of action for *Npas2* downregulation may be unknown, yet the drug screening conducted showed significant *Npas2* downregulation for each of these compounds, making their role in the circadian cycle an avenue for future research.

Denoted as being trimeric ECM proteins due to being comprised of three protein subunits (15), collagen types I and III are the primary components in skin ECM due to their ability to form thicker fibers (16). Moreover, these collagen fibers have been shown to change their orientation within the skin in response to injury, leading to HTS formation (17). In normal skin, collagen maintains a set ratio between protein content, but HTS results in increased expression of types I and III mRNA and protein as compared with normal skin (30). In this study, Dwn1 at dose of 10 μM showed attenuated gene expression of collagen types I and III on HDFa (Figure 2A), suggesting Dwn1 regulates the attenuation of fibrosis. While Dwn2 was shown to mitigate the total collagen deposition, results from Figure 2A indicate the lack of significant downregulation of collagen synthesis genes by Dwn2. This result may be attributed to the scope of this study, as it was limited to collagen types 1 and 3 due to the involvement of those specific proteins in the formation of HTS. It is possible that Dwn2 was responsible for downregulating gross collagen deposition as a result of mitigation to gene activity levels for collagen proteins outside the scope of this study. In addition, myofibroblasts, as the effector cells, mainly differentiated from fibroblasts, play the crucial role in the pathophysiology of HTS (31). TGFb1, an important profibrotic cytokine, induces the differentiation of myofibroblasts expressing a-SMA (32). In this study, the myofibroblast marker, α-SMA were significantly inhibited on the TGFb1-activated cells treated with Dwn1 and Dwn2 (Figure 2B). Dwn1 and Dwn2 may contribute to wound healing by preventing hypertrophic scarring.

**FIGURE 3:**
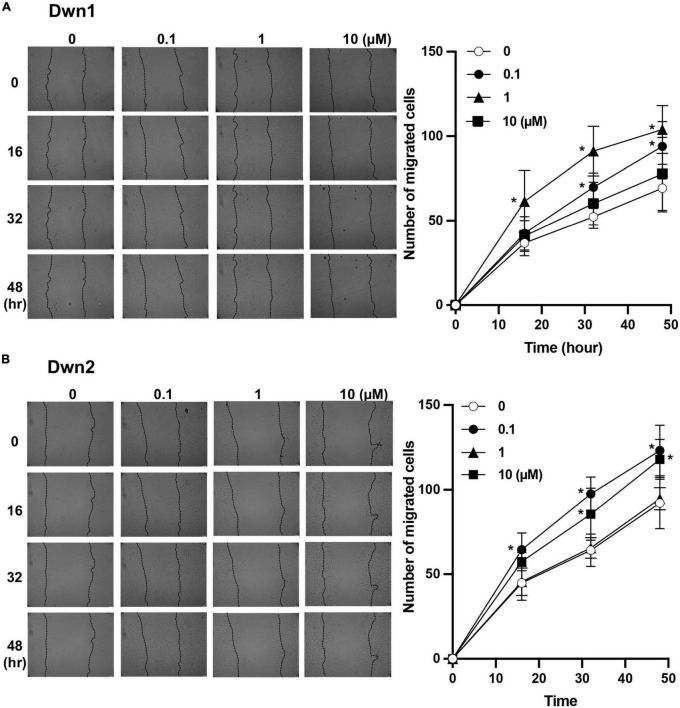
Cell migratory assay. **(A)** The number of migratory cells was recorded throughout 48-h treatment for both control (0 μM) and Dwn1 treated groups. The lefthand image shows images of scratch test site with cell infiltration used to generate graph. The average number of Dwn1 treated cells indicated significantly higher cell migration beyond the scratch test border for all time points in the 0.1 and 1 μM treatment groups. **(B)** The number of migratory cells was recorded throughout 48-h treatment for both control and Dwn2 (0 μM) treated groups. The lefthand image shows images of scratch test site with cell infiltration used to generate graph. The average number of Dwn2 treated cells indicated significantly higher cell migration beyond the scratch test border for all time points in the 0.1 and 10 μM treatment groups (*N* = 6). The *p*-value was calculated between these groups (*p < 0.05). Graphs represent the average ± STD.

Scratch tests conducted on fibroblast monolayers showed higher cell mobility during the active phase of cellular activity. This coincides with the results established by Figure 3, where higher rates of cell mobility indicate a faster rate of wound closure and thus wound healing. The ability for cells to infiltrate the wound area more quickly after initial injury has been shown to not only quicken the recovery process (16) but can more closely regulate the amount of collagen deposited into the wound site, thereby preventing the excessive deposition of collagen proteins (8). As such, Dwn1 and Dwn2 seem to be promising candidates for clinical applications due to the ability to regulate gene expression levels throughout multiple days in the wound healing process. Moreover, this work demonstrates the ability of Dwn1 and Dwn2 to attenuate the activity of fibroblasts without causing cell death. The results from Figure 1B demonstrate this phenomenon, as there was a high cell viability for each of the hit compounds. As such, this study offers insights to how the regulation of these cell types can be influenced by hit compound treatment, thus supporting the ability of these compounds to be useful in a clinical setting.

Although the study set out to determine the ability of five different compounds to regulate *Npas2* activity, only Dwn1 and Dwn2 emerged as being able to significantly mitigate overall collagen biosynthesis through regulation of collagen synthesis genes (Figure 2). As such, these compounds offer the possibility to serve as therapeutic agents for dermal fibroblasts. The other three molecules did not exhibit sufficient downregulation of collagen, thereby preventing them from being used as possible treatment or prevention for HTS in a clinical setting. Although the exact cell signaling pathway taken by either of these two compounds is currently unknown, results from these experiments set forth the possibility for these two compounds to follow a similar pathway within the cell. This study was limited in scope due to resource constraints. As such, the selection of hit compounds outside of the scope of this study may prove beneficial for further investigation and serve as an avenue for continued research. Furthermore, this study may be optimized in the future to include *in vivo* testing of Dwn1 and Dwn2 to reveal optimal drug concentrations and application frequencies to combat the formation of hypertrophic scarring. Future directions for this research include *in vivo* testing of animal models. In addition, further studies could be used to identify the specific pathways used by Dwn1 and Dwn2 to alter gene activity, thereby providing insights into a possible signal cascade able to be utilized for more extensive wound healing studies.

## Conclusion

In this study, we demonstrated that *NPAS2* suppression in HDFa by compounds had an effect on collagen deposition, collagen-related gene expression, and myofibroblast differentiation without cytotoxicity. The results of this study suggest that Dwn1 and Dwn2 could be novel therapeutic agents capable of promoting collagen homeostasis and accelerating wound healing with minimal hypertrophic scarring.

## Data availability statement

The raw data supporting the conclusions of this article will be made available by the authors, without undue reservation.

## Author contributions

AH, IN, and RJ contributed to the study design and funding acquisition. AC, YS, AH, ZB, YR, and TK contributed to the data collection and statistical analysis. YS and AH contributed to the data interpretation and critical analysis. AC and AH contributed to the drafting of the manuscript. IN and RJ contributed to the critical revision of the manuscript. All authors contributed to the article and approved the submitted version.
