# Supplementary figures and images for: In vitro assessment of Neuronal PAS domain 2 mitigating compounds for scarless wound healing
Source: Front Med (Lausanne). 2023 Feb 1;9:1014763. doi: 10.3389/fmed.2022.1014763 (PMC9928850; doi:10.3389/fmed.2022.1014763)

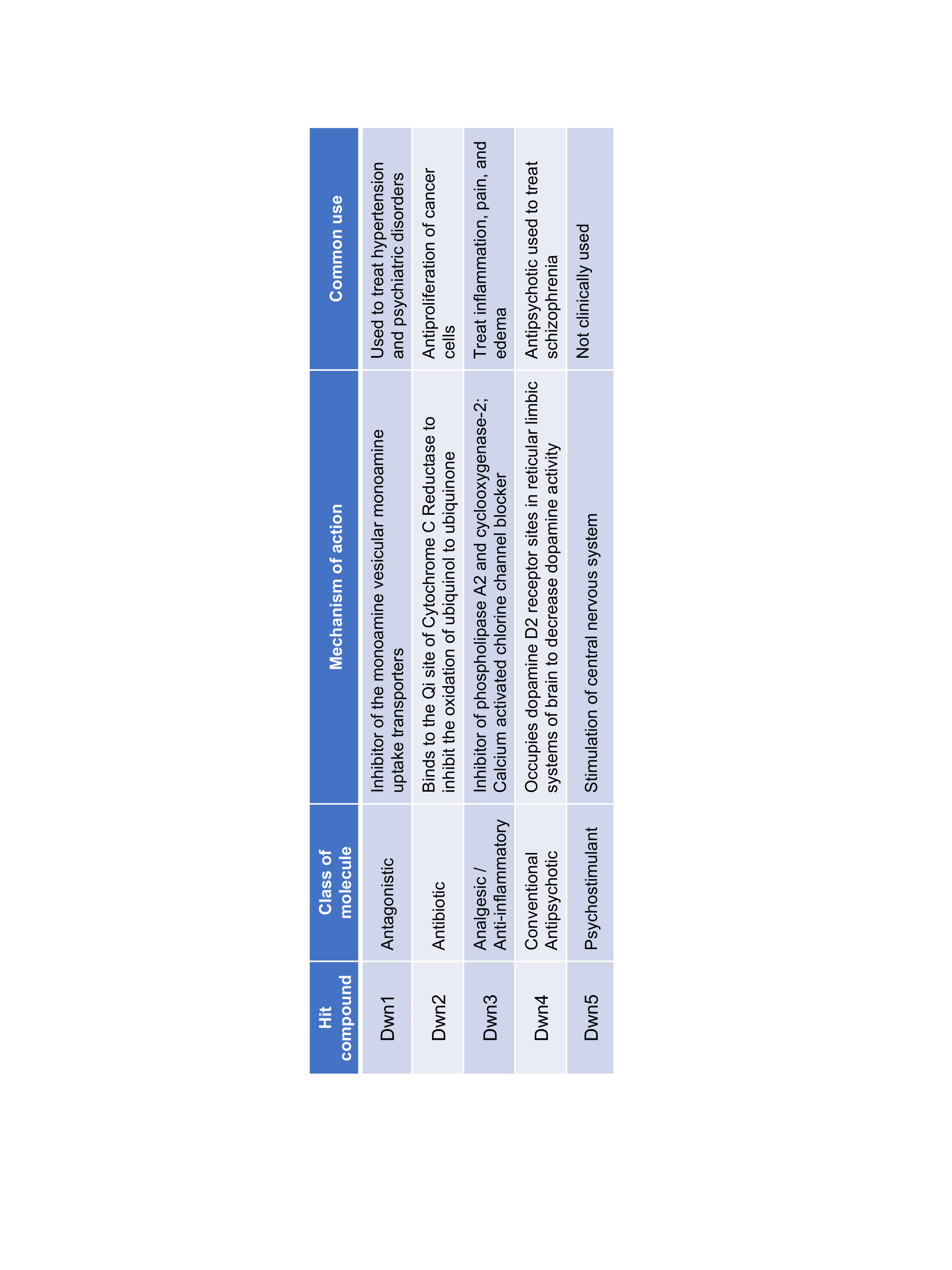

Supplement: Supplementary file 1 [file Image_1.JPEG]
